# Japanese spotted fever in an area endemic to SFTS virus: Case report and review of the literature

**DOI:** 10.1097/MD.0000000000039268

**Published:** 2024-08-09

**Authors:** Su-nan Liu, Wei Li

**Affiliations:** a Department of Emergency, Union Hospital, Tongji Medical College, Huazhong University of Science and Technology, Wuhan, Hubei, China; b Department of Infectious Diseases, Union Hospital, Tongji Medical College, Huazhong University of Science and Technology, Wuhan, Hubei, China.

**Keywords:** case report, Japanese spotted fever, *Rickettsia japonica*, severe fever with thrombocytopenia syndrome, spotted fever group rickettsioses

## Abstract

**Rationale:**

The geographic spread of Japanese spotted fever (JSF) in China is gradually expanding, particularly in regions where severe fever with thrombocytopenia syndrome (SFTS) is highly prevalent, with both diseases sharing similarities in epidemiology and clinical presentation. The microbiological diagnosis of JSF is challenging, compounded by low awareness among healthcare professionals in newly affected areas. Moreover, primary healthcare facilities without polymerase chain reaction (PCR) testing capabilities for SFTS often misdiagnose JSF as SFTS.

**Patient concerns:**

All 3 patients had a history of working in the fields, with cold like symptoms in the early fever stages, but the fever did not improve after a few days. The accompanying symptoms were also very different. Physical examination revealed enlarged lymph nodes, different forms of rash, with or without eschar. Laboratory tests showed thrombocytopenia, eosinophilia, elevated lactate dehydrogenase, and transaminase, with 1 patient experiencing renal damage. It is worth noting that these 3 patients reside in an area where SFTS is endemic, and there have been no prior reports of JSF. They exhibited clinical symptoms and laboratory test results closely resembling those of SFTS. Therefore, they were initially misdiagnosed with SFTS in their local hospitals.

**Diagnoses:**

The 3 patients who arrived at our hospital 7 days after symptom onset and were subsequently diagnosed with JSF by metagenomic next-generation sequencing (mNGS).

**Interventions:**

Doxycycline treatment for 1 week.

**Outcomes:**

The patients’ symptoms quickly improved with no side effects, and the results of laboratory tests went back to normal.

**Lessons:**

By comparing the clinical characteristics of JSF patients and SFTS patients comprehensively, we found that APTT and procalcitonin levels may be valuable in assisting in the identification of SFTS and JSF. In all areas where tick-borne diseases are endemic, include SFTS-epidemic areas, we recommend using the Weil–Felix test to screen for potential rickettsiosis in patients presenting with fever and thrombocytopenia with or without rash in primary healthcare settings, as well as simultaneous testing for the SFTS virus and spotted fever group rickettsioses sequence. Additionally, mNGS sequencing should be used to confirm the diagnosis and provide information for epidemiological investigations in patients who are suspected of having spotted fever group rickettsiosis.

## 1. Introduction

The human spotted fever group rickettsioses (SFGR) endemic is spreading globally^[1]^ with reports of *Rickettsia japonica* (*R. japonica*) infection emerging in China since 2013,^[2]^ indicating the disease’s gradual spread from central and coastal areas to western regions.^[2–8]^ Nevertheless, in newly affected areas, primary care physicians have limited awareness of this disease.

The tick species responsible for hosting *R. japonica* and transmitting the severe fever with thrombocytopenia syndrome virus (SFTSV) are *Haemaphysalis longicornis* (*H. longicornis*).^[9,10]^ In the eastern and central regions of China, *H. longicornis* is a predominant tick species. Consequently, the seasonal and geographical distribution of *R. japonica*, the pathogen of Japanese spotted fever (JSF), coincides with that of severe fever with thrombocytopenia syndrome (SFTS), a locally prevalent febrile infectious disease. Given the striking similarities between these 2 diseases, misdiagnosis of JSF as SFTS, or delayed diagnosis, can result in disease exacerbation, multi-organ failure, and even mortality, necessitating intensive care unit admission, ventilator support, and blood purification, which further exacerbates the disease burden.

The importance of early diagnosis for the prompt initiation of doxycycline antibiotic treatment cannot be overstated. However, the etiological diagnosis of SFGR faces certain challenges. When a patient is suspected of having rickettsial disease, due to species diversity and differing regional distribution, it is difficult to predict and select a specific detection method that covers all possible species in the genus *Rickettsia.* As a highly pathogenic microorganism, SFGR resides primarily within endothelial cells lining blood vessels, with only a few circulating pathogens, making techniques such as polymerase chain reaction (PCR) and metagenomic next-generation sequencing (mNGS) susceptible to false-negative results. Antibodies against SFGR typically appear 7 to 14 days after symptom onset, with IgM persisting for 3 to 4 months. However, antibody cross-reactivity between *R. japonica* and *Rickettsia typhi* has been reported.^[11]^ Moreover, hospitals in newly endemic areas do not have specific antibody reagents on hand. As a result, the early diagnostic efficacy of serology tests is reduced, as is the interpretation of positive results. Isolation and cultivation of rickettsial pathogens is also difficult, because a biosafety level of 3 or higher is a laboratory requirement. Taken together, we need to identify key differentiation points between JSF and SFTS during the early stages based on clinical characteristics and routine diagnostic tests.

In this report, 3 cases of JSF in the Hubei STFS endemic area were introduced. Two of them were from Huanggang, Hubei, which we reported in 2021,^[5]^ and the other was the first report of JSF from Enshi, Hubei, which we treated in August of 2023. In their local hospitals, all 3 patients were initially misdiagnosed with SFTS. After admission to Wuhan Union Hospital (Wuhan, Hubei, China) on the seventh day after onset, they were finally diagnosed with JSF by mNGS. We compared these 3 JSF cases with 12 SFTS cases at 5 to 8 days of morbidity to identify key differential diagnostic clinical characteristics and laboratory tests for JSF at various levels of clinical settings in STFS endemic areas.

## 2. Case report

The study is retrospective and involves no additional specimen collection or medical intervention; therefore, ethical approval was waived.

### 2.1. Case description

#### 2.1.1. Patient No. 1

This 63-year-old female patient was admitted to the hospital in August 2023 due to a week of continuous fever and a generalized rash that had appeared over the previous 5 days (Table 1). At the onset of symptoms, she was living in an urban building near mountainous terrain in Lichuan County, Enshi City, Hubei Province. She once visited a local farmers’ field for agritourism, where she picked fresh edamame beans.

**Table 1:** Epidemiologic and characteristics of the 3 JSF patients.

Characteristics	Patient No.		
	1	2	3
Age (yr)	63	62	59
Sex	F	M	F
Aware of tick bite	No	No	Yes
Field work	Yes	Yes	Yes
Time from tick bite to disease onset (d)	NA	NA	2
Time from disease onset to hospital admission (d)	7	7	7
No. days hospitalization	7	9	7
Place of residence	Lichuan, Hubei	Huanggang, Hubei	Huanggang, Hubei

NA = not available.

#### 2.1.2. Patient Nos 2 and 3

Two patients from Macheng City, Huanggang City, Hubei Province, specifically from the hilly area of Fuzihe Town, sought medical attention in August 2021.^[5]^ They presented with symptoms including fever, fatigue, severe headache, and rash. One patient was a 62-year-old male, and the other was a 59-year-old female.

All 3 patients were hospitalized in the Department of Infectious Diseases at Union Hospital, affiliated with Tongji Medical College at Huazhong University of Science and Technology.

### 2.2. Comparison of clinical characteristics and laboratory results of 3 JSF patients with those of 12 SFTS patients

**Figure 1:**
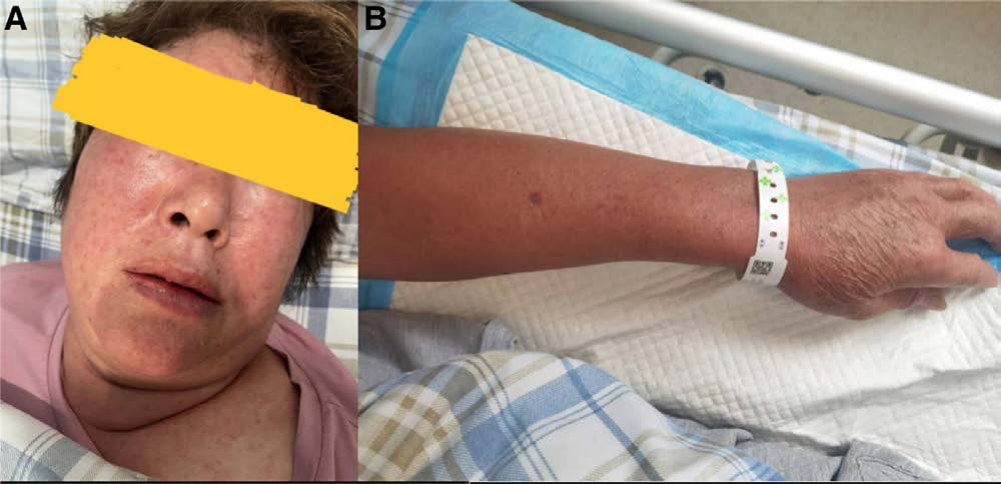
(A) Patient No. 1 has a widespread rash covering the entire body, including the head and face, appearing bright red; (B) bruises are present on Patient No. 1’s arms.

**Figure 2.**
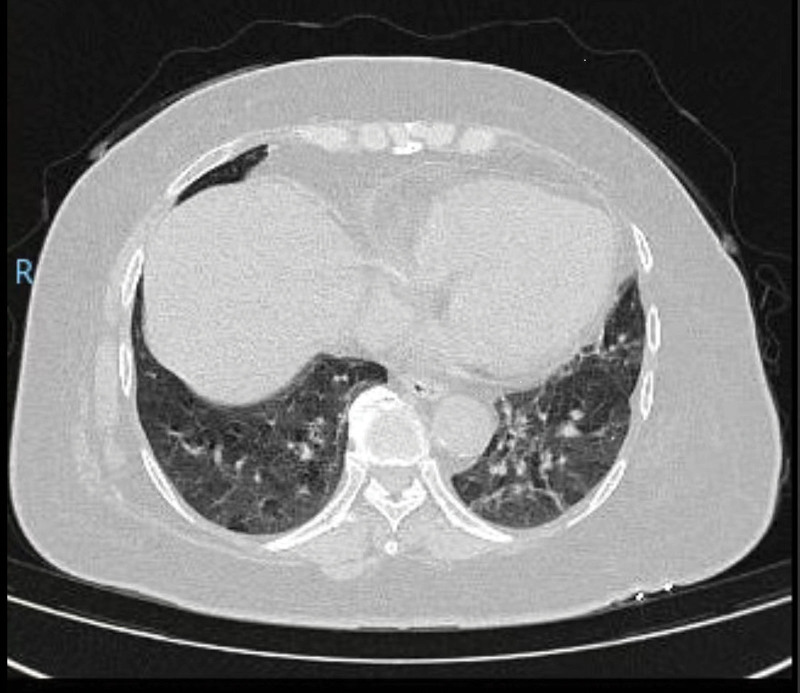
The pulmonary and mediastinal 3-dimensional CT scans of Patient No. 1 revealed scattered inflammatory lesions or subsegmental atelectasis in both lungs.

Patient Nos 2 and 3 had the same potential JSF contact history as Patient No. 1, as they all had a history of gardening in a vegetable patch 10 days prior to symptom onset, and Patient Nos 2 and 3 were daily field laborers. As shown in Figure 1A, Patient No. 1 developed a widespread bright red rash covering her entire body, including head and face. The presence of bruises on Patient No. 1’s arms is depicted in Figure 1B. In contrast, Patient Nos 2 and 3 developed dark rashes that went unnoticed at first due to their darker skin tone, which is common among farmers, and were only discovered after a thorough physical examination, with eschars only visible on the surface of Patient No. 3’s body.^[5]^ Additionally, Patient No. 1 had significant nausea and vomiting but no obvious headaches or muscle pain. Although she had no respiratory symptoms such as coughing, sputum production, or wheezing, her pulmonary and mediastinal 3-dimensional CT scans revealed scattered inflammatory lesions or subsegmental atelectasis in both lungs (Fig. 2). To identify the distinguishing characteristics, we compared the clinical features (Table 2) and laboratory results (Table 3) of these 3 JSF patients to those of 12 confirmed cases of SFTS patients aged 55 to 65 years who experienced the onset of illness within 5 to 8 days.

**Table 2:** Clinical characteristics of 3 JSF patients and 12 SFTS patients.

Signs and symptoms	JSF Patient No.			12 SFTS patients
	1	2	3	
Fever	Yes	Yes	Yes	12/12
Highest temperature (°C)	40.1	39.1	40.2	38.5–40.1
Headache	No	Yes	Yes	7/12
Asthewenia	Yes	Yes	Yes	12/12
Masseter weakness	No	Yes	No	8/12
Myalgia	No	No	Yes	7/12
Anorexia	Yes	Yes	Yes	10/12
Nausea and vomiting	Yes	No	No	4/10
Cough	No	No	No	0/12
Confusion	Yes	No	No	5/12
Meningeal irritation sign	No	No	No	0/12
Ecchymosis	Yes	No	No	11/12
Rash	Yes	Yes	Yes	0/12
Eschar	No	No	Yes	0/12
Lymphadenopathy	Yes	Yes	Yes	12/12

**Table 3:** Routine laboratory results of 3 JSF patients and 12 SFTS patients.

Result	Normal range	JSF Patient No.			12 SFTS patients median (range) Day 5 to 8 after onset
		1	2*	3*	
WBC count (×10^9^/L)	3.5–9.5	11.48	7.53	6.78	2.21 (0.99–3.27)
N%	40–75	93.0	74.6	86.5	56.6 (35.2–71.4)
E%	0.4–8.0	0	0	0	0.03 (0–0.20)
L%	20–50	3.8	17.6	12.2	12.1 (9.2–21.5)
Thrombocytopenia (×10^9^/L)	125–350	79	89	113	35 (17–64)
Proteinuria	Negative	Positive	Positive	Negative	11/12 positive
Hematuria	Negative	Positive	Positive	Negative	12/12 positive
Total bilirubin (μmol/L)	5.1–19.0	16.5	7.7	9.6	15.45 (8.2–55.2)
APTT (s)	28.0–43.5	37.6	26.5	32.9	58.7 (48.5–70.4)
ALT (U/L)	5–40	61	95	41	115 (56–366)
AST (U/L)	8–40	58	99	29	152 (79–275)
LDH (U/L)	109–245	477	498	319	872 (298–3245)
D-dimer (mg/L)	<0.5	0.97	4.51	2.32	1.21 (0.46–6.49)
PCT (μg/L)	<0.5	4.82	12.2	0.68	0.11 (<0.05–0.39)
CRP (mg/L)	<8.00	318.7	105.0	83.1	37 (<8–57)
Cr (μmol/L)	44.0–133.0	72.5	49.8	86.2	69 (52–127)

ALT = alanine aminotransferase, APTT = activated partial thromboplastin time, AST = aspartate aminotransferase, Cr = creatinine, E% = eosinophils percentage, L% = lymphocyte percentage, LDH = lactate dehydrogenase, N% = neutrophil percentage, PCT = procalcitonin levels, WBC = white blood count.

### 2.3. Molecular diagnosis of JSF

**Figure 3.**
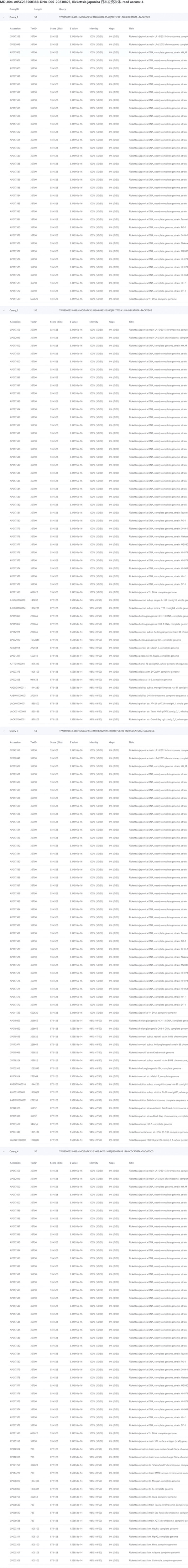
Blast comparison results of the 4 *R. japonica* 50-bp sequences detected in Patient No. 1.

The pathogens detected by mNGS in samples obtained from Patient No. 3 are listed in Table 4. In addition to common human symbiotic microorganisms, spotted fever rickettsiosis was identified in the specimen as 4 read fragments aligned with 100% identity to the *R. japonica* reference sequences (Fig. 3), which is consistent with the mNGS results of Patient Nos 2 and 3.^[5]^ It was not possible to complete the gene sequence analysis due to the limited amount of fragments found.

**Table 4:** Pathogenic microorganisms detected by mNGS in blood (sorted by species).

Patient No.	Name	Genus name	Genus reads accum
1	*Rickettsia*	*Rickettsia*	29
	Spotted fever group	*Rickettsia*	17
	*Rickettsia japonica*	*Rickettsia*	4
	*Delftia*	*Delftia*	1
	*Delftia tsuruhatensis*	*Delftia*	1
2	*Staphylococcus*	*Staphylococcus*	4
	*Staphylococcus aureus*	*Staphylococcus*	4
	*Staphylococcus capitis*	*Staphylococcus*	4
	*Rickettsia*	*Rickettsia*	2
	Spotted fever group	*Rickettsia*	2
	*Cutibacterium*	*Cutibacterium*	1
	*Cutibacterium acnes*	*Cutibacterium*	1
	Herpesviridae	Herpesviridae	14
	Gammaherpesvirinae	Gammaherpesvirinae	14
	*Lymphocryptovirus*	*Lymphocryptovirus*	14
	*Human gammaherpesvirus 4*	*Lymphocryptovirus*	14
3	*Moraxella*	*Moraxella*	18
	*Moraxella osloensis*	*Moraxella*	18
	*Cutibacterium*	*Cutibacterium*	10
	*Cutibacterium acnes*	*Cutibacterium*	10
	*Rickettsia*	*Rickettsia*	4
	Spotted fever group	*Rickettsia*	4
	*Staphylococcus*	*Staphylococcus*	2
	*Staphylococcus aureus*	*Staphylococcus*	2
	*Staphylococcus capitis*	*Staphylococcus*	2
	*Malassezia*	*Malassezia*	1
	*Malassezia restricta*	*Malassezia*	1
	Herpesviridae	Herpesviridae	2
	*Alphaherpesvirinae*	*Alphaherpesvirinae*	1
	*Simplexvirus*	*Simplexvirus*	1
	*Human Alphaherpesvirus 1*	*Simplexvirus*	1
	*Betaherpesvirinae*	*Betaherpesvirinae*	1
	*Cytomegalovirus*	*Cytomegalovirus*	1
	*Human Betaherpesvirus 5* (*Cytomegalovirus*)	*Cytomegalovirus*	1

### 2.4. Therapeutic responses

Following doxycycline treatment, the patient’s symptoms quickly improved with no side effects, and the results of laboratory tests went back to normal.

## 3. Discussion

The tick-borne SFGR has been widely recognized as a significant emerging zoonotic group of diseases worldwide. JSF, a newly emerging SFGR, was discovered in Japan in 1984, with cases occurring between April and October of that year. The causative agent was identified as *R. japonica*.^[9]^ Uchida et al^[12]^ demonstrated that the arthropod reservoirs *H. longicornis* serve as the vector for JSF transmission, because immunofluorescence testing with monoclonal antibodies specific to *R. japonica* confirmed the presence of rod-shaped organisms in the hemolymph preparations of *H. longicornis*. *H. longicornis* is also widely distributed in China and is the most common tick species found near human habitats, with cattle and sheep serving as the primary hosts.^[13]^ In Asia, *H. longicornis* ticks have been reported to be infected with SFTSV, spotted fever group rickettsioses, *Anaplasma* species, *Borrelia burgdorferi sensu lato*, and *Babesia* species, with the specific pathogens varying by region.^[10,14]^ *H. longicornis* is active from April to October each year, which coincides with the peak of human outdoor activities and the prevalence of SFTS.

Cases of JSF have been reported in various regions in China, including Liuan in Anhui,^[2]^ Xinyang in Henan,^[3]^ Lin’an in Zhejiang,^[4]^ Huanggang,^[5]^ and Zigui in Hubei,^[6]^ as well as individual cases reported in Shangluo in Shaanxi^[7]^ and Guangyuan in Sichuan, all between April and October.^[8]^ Here, we present the first JSF case reported in Enshi. In JSF endemic areas, *H. longicornis*, in which *R. japonica* has been detected, is the predominant tick species.^[2,15]^ In China, *H. longicornis* may serve as the primary host and vector for *R. japonica*. SFGR can be transmitted through tick bites at all developmental stages (larvae, nymphs, and adults).^[16,17]^ Tick bites are painless, and tick nymphs and larvae are so small that they are difficult to detect. As a result, all 3 JSF patients denied being bitten by ticks during our inquiries, but this should not be used as the sole criterion for excluding SFGR. According to reported cases in China, SFGR has spread from the Tianmu Mountains, Dabie Mountains, and Daba Mountains in the east to the Qinling Mountains and Wuling Mountains in the west.^[2–8]^ Although there have been no reported cases of JSF in the eastern coastal regions of Shanghai and Shandong, *R. japonica* has been identified in *H. longicornis* nymphs and adults found in the local environment.^[18,19]^ The increasing number of JSF patients discovered suggests that a complete JSF transmission chain has gradually formed throughout China. Due to similarities in latitude, climatic conditions, the geographical environment, and the widespread presence of *H. longicornis* ticks, certain regions in China may experience a seasonal epidemic trend of JSF in the future, like in Japan. Although the total number of confirmed cases reported is not very high, the difficulties in pathogen diagnosis and the presence of an unknown number of atypical mild cases suggest that the actual number of infected individuals may be higher, which indicates the need to prepare for an increasing number of cases requiring medical attention.

All 3 JSF patients were from SFTS-endemic areas in Hubei, and were admitted to our hospital on the 7th day after the onset of the disease. Common symptoms included fever at onset, asthenia, anorexia, lymphadenopathy, and the appearance of a generalized red maculopapular rash 2 days after fever onset. Laboratory examinations for all 3 patients revealed a complete absence of eosinophils, decreased platelet counts, elevated ALT levels, increased lactate dehydrogenase levels, and elevated D-dimer levels. Additionally, 2 of the patients exhibited proteinuria and hematuria, which closely resemble characteristics of SFTS. The presence of *R. japonica* was confirmed by mNGS results from all 3 patients’ samples, and the diagnosis of SFTS was ruled out at the same time. *R. japonica* can cause occlusive thrombosis and ischemic necrosis at the site of invasion, as well as edema and ischemic organ damage due to systemic vascular infection and platelet consumption. SFTS belongs to viral hemorrhagic fevers and targets endothelial cells in a manner similar to *R*. *japonica*. The virus disrupts the endothelial system of microvessels, leading to microvascular damage. Both diseases share many similarities in pathogenesis and pathology. Through a comparison of clinical presentations between these 3 JSF patients and 12 SFTS patients who became ill 5 to 8 days later, we found that symptoms observed in SFTS cases could also occur in JSF. The 3 JSF patients developed a rash 2 or 3 days after symptoms began. Subsequent investigation into the patient’s medical background revealed that the rash that manifested a number of days subsequent to the initiation of symptoms was erroneously attributed to measles, syphilis, or a drug reaction. On the other hand, there are also JSF patients who do not develop a rash.^[3,4,20]^ In addition, rashes in patients with JSF are nonpruritic; in some cases, the rash appears darker in color. Furthermore, JSF patients engaged in agricultural work may have darker skin tones, potentially making the rash less noticeable. The STFS patients did not exhibit eschars, but one of these 3 JSF patients did. According to reports, severe JSF cases can exhibit symptoms such as disseminated intravascular coagulation, multi-organ failure, mental disorders, aseptic meningitis, and acute respiratory distress syndrome.^[21,22]^ These clinical manifestations are also difficult to differentiate from those associated with severe SFTS.^[23]^

The pathogen of SFGR belongs to intracellular parasitic gram-negative microorganisms with pathogenic components that resemble bacterial endotoxins. Therefore, on the 7th day after symptom onset, we observed an elevation in procalcitonin levels and an increased neutrophil ratio, which is consistent with other reports.^[24]^ In contrast, SFTS, as a viral infection, does not lead to an increase in procalcitonin levels unless there is a secondary bacterial or fungal infection. In SFTS-endemic areas, JSF or SFTS patients are initially treated with quinolones or penicillin for nonspecific flu-like symptoms, and if there is no improvement, they typically seek care at comprehensive hospitals around 5 to 7 days after disease onset. During this period, procalcitonin levels can serve as a laboratory indicator for distinguishing between JSF and SFTS.

Procalcitonin testing may not be available in most basic healthcare facilities. The Weil–Felix test involves a nonspecific agglutination reaction against Proteus OX19, a bacterium with shared antigens with *Rickettsia* species. It can be used to detect the presence of *Rickettsia* antibodies in a patient’s serum, and the Weil–Felix test may yield positive results during the first week of JSF.^[2]^ However, comprehensive hospitals have discontinued this test due to its low sensitivity and specificity, which may produce false positives in conditions such as spotted fever, endemic typhus, typhoid, and brucellosis. Therefore, we believe the Weil–Felix test can still be employed as a screening test for rickettsial diseases in primary healthcare facilities because it is cost-effective and easy to perform, especially in endemic areas, and can assist doctors in distinguishing between rickettsial diseases and SFTS. Furthermore, in addition to the presence of eschars, positive detection results can indicate the possibility of a rickettsial infection and may serve as an early indication for the use of doxycycline.

mNGS can detect various pathogens in the blood and provide differential diagnoses for multiple pathogenic infections, but it is relatively expensive and its platforms are typically located in large- to medium-sized cities. Transporting specimens for mNGS can thus be difficult for patients living in remote mountainous areas. In recent years, we have observed several cases of patients presenting with fever, skin rashes, and ulcerative lesions, suggestive of rickettsial diseases, but mNGS testing came back negative. These patients recovered after receiving doxycycline treatment. This could be attributed to the fact that because rickettsiae primarily infect endothelial cells, resulting in low pathogen levels in the bloodstream, particularly in mild cases, mNGS testing may fail to detect rickettsiae. Nevertheless, mNGS testing can provide a confirmed diagnosis and molecular identification for *Rickettsia*-positive samples, especially in the case of the first infected individual in the region. Further whole-genome sequencing by disease control authorities will contribute to epidemiological investigations, which will facilitate timely dissemination of early warning information to healthcare institutions in the areas where cases have been identified.

## 4. Materials and methods

### 4.1. Routine clinical laboratory tests

Peripheral blood samples were collected from each patient according to standard procedures during admission, and procalcitonin (PCT) levels, C-reactive protein levels, D-dimer, urinary sediment microscopy, and differential blood cell counts were examined at the Clinical Diagnosis Laboratory of Union Hospital. Plasma levels of creatinine, alanine aminotransferase, aspartate aminotransferase, and lactate dehydrogenase were measured at the Biochemistry Laboratory of Union Hospital. Furthermore, the activated partial thromboplastin time of fresh plasma samples was measured at the Clinical Diagnosis Laboratory of Union Hospital.

### 4.2. mNGS tests

#### 4.2.1. Library preparation and metagenomic sequencing

To separate the plasma for DNA sequencing, whole blood was centrifuged at 1600 g for 10 minutes, and the supernatant was further centrifuged at 16,000 g for 10 minutes. The mNGS library was then prepared by automatic plasma nucleic acid extraction, enzymatic fragmentation, end repair, terminal adenylation, and adaptor ligation, according to the methods reported in a previous study.^[25]^ Bead-purified libraries quantified by real-time PCR (KAPA) were pooled for shotgun sequencing on the Illumina NextSeq. Approximately 20 million 50-bp single-end reads were generated for each library, and a bioinformatic analysis was conducted as described in a previous report.^[26]^ Human origin sequences (GRCh38.p13) were filtered, and the remaining reads were aligned to a reference database (NCBI nt, GenBank, and in-house curated genomic database) to identify the microbial species and read count. For each sequencing run, a negative control (NC; culture medium containing 10^4^ Jurkat cells/mL) was included.

#### 4.2.2. mNGS reporting criteria

Microbial reads identified from a library were reported if: The sequencing data passed quality control filters (library concentration > 50 pM, Q20 > 85%, Q30 > 80%); The NC in the same sequencing run did not contain the species or an RPM (sample)/RPM (NC) ≥ 5, which was determined empirically according to previous studies^[25,27,28]^ as a cut off for discriminating true-positives from background contaminations.

## 5. Conclusions

JSF exhibits a spreading and disseminating trend; therefore, in newly affected regions, local disease control departments should notify healthcare institutions at all levels within their jurisdiction about the first confirmed case as a warning signal, thereby increasing local physicians’ awareness of the presence of the disease. The Weil–Felix test can be employed as an auxiliary diagnostic tool for rickettsial diseases in primary healthcare settings. In prevalent regions, due to the similarity in vectors and clinical features between SFTS and JSF, it is crucial to differentiate between them. Within the first week of onset, procalcitonin is a recommended discriminative indicator, urging early initiation of doxycycline treatment. APTT may also be helpful. mNGS testing on suspected SFGR patients is of significant value in confirming pathogenic *Rickettsia* infection, and its results can also be used to guide epidemiological investigations. Especially in the case of the first confirmed diagnosis of JSF in a particular locality, mNGS can provide an early warning for local healthcare providers, enhance awareness, and potentially increase the likelihood of early diagnosis and treatment for subsequent cases. Additionally, in endemic areas, patients with suspected SFTS symptoms who are negative for the SFTSV PCR test should be considered for SFGRs such as JSF, and empirical treatment with doxycycline should be initiated as soon as possible. The limitation of this study is that *R. japonica*, a highly pathogenic microorganism, primarily infects endothelial cells intracellularly, resulting in low pathogen levels in the patient’s blood. We did not conduct whole-genome sequencing of the pathogen to compare it to prevalent strains from other regions, which limited our ability to determine its origin and evolution. As a result, we could not provide deeper insights into the microbiological and epidemiological aspects. Future studies would benefit from larger sample sizes and biopsy specimens, allowing for more comprehensive analysis and deeper insights into relevant research data.

## Author contributions

**Data curation:** Su-nan Liu.

**Formal analysis:** Su-nan Liu.

**Writing – original draft:** Su-nan Liu.

**Writing – review & editing:** Wei Li.
